# Projecting prevalence, costs and evaluating simulated interventions for diabetic end stage renal disease in a Canadian population of aboriginal and non-aboriginal people: an agent based approach

**DOI:** 10.1186/s12882-017-0699-y

**Published:** 2017-09-04

**Authors:** Amy Gao, Nathaniel D. Osgood, Ying Jiang, Roland F. Dyck

**Affiliations:** 1 Strategic Planning and Data Warehousing, University of Alberta, Edmonton, Canada; 2 Department of Computer Science, University of Saskatchewan, Saskatoon, Canada; 3 Cancer Care Ontario, Toronto, ON Canada; 4 Department of Medicine (Canadian Center for Health and Safety in Agriculture), University of Saskatchewan, Saskatoon, Canada

**Keywords:** Diabetes, End stage renal disease, Computer model, Agent based, Projection, Costs, Aboriginal, Indigenous, Epidemiology

## Abstract

**Background:**

Diabetes-related end stage renal disease (DM-ESRD) is a devastating consequence of the type 2 diabetes epidemic, both of which disproportionately affect Indigenous peoples. Projecting case numbers and costs into future decades would help to predict resource requirements, and simulating hypothetical interventions could guide the choice of best practices to mitigate current trends.

**Methods:**

An agent based model (ABM) was built to forecast First Nations and non-First Nations cases of DM-ESRD in Saskatchewan from 1980 to 2025 and to simulate two hypothetical interventions. The model was parameterized with data from the Canadian Institute for Health Information, Saskatchewan Health Administrative Databases, the Canadian Organ Replacement Register, published studies and expert judgement. Input parameters without data sources were estimated through model calibration. The model incorporated key patient characteristics, stages of diabetes and chronic kidney disease, renal replacement therapies, the kidney transplant assessment and waiting list processes, costs associated with treatment options, and death. We used this model to simulate two interventions: 1) No new cases of diabetes after 2005 and 2) Pre-emptive renal transplants carried out on all diabetic persons with new ESRD.

**Results:**

There was a close match between empirical data and model output. Going forward, both incidence and prevalence cases of DM-ESRD approximately doubled from 2010 to 2025, with 250–300 new cases per year and almost 1300 people requiring RRT by 2025. Prevalent cases of First Nations people with DM-ESRD increased from 19% to 27% of total DM-ESRD numbers from 1990 to 2025. The trend in yearly costs paralleled the prevalent DM-ESRD case count. For Scenario 1, despite eliminating diabetes incident cases after 2005, prevalent cases of DM-ESRD continued to rise until 2019 before slowly declining. When all DM-ESRD incident cases received a pre-emptive renal transplant (scenario 2), a substantial increase in DM-ESRD prevalence occurred reflecting higher survival, but total costs decreased reflecting the economic advantage of renal transplantation.

**Conclusions:**

This ABM can forecast numbers and costs of DM-ESRD in Saskatchewan and be modified for application in other jurisdictions. This can aid in resource planning and be used by policy makers to evaluate different interventions in a safe and economical manner.

**Electronic supplementary material:**

The online version of this article (10.1186/s12882-017-0699-y) contains supplementary material, which is available to authorized users.

## Background

A pandemic of type 2 diabetes (T2DM) is affecting diverse populations worldwide [1]. While genetic factors are important precursors [2, 3], the rapid emergence of T2DM since the middle of the last century parallels a rise in obesity rates associated with unprecedented lifestyle changes affecting caloric intake and physical activity [1, 3]. In addition, growing numbers of people with diabetes at each end of the life span attest to other contributing factors. Thus, T2DM during childhood and early adulthood is often related to gestational diabetes, which heightens diabetes risk for both affected mothers [4] and their offspring [5, 6]. Among aging adults, increasing numbers of baby boomers entering their seventh decade, and improved survival of people with diabetes, are also driving an increase in T2DM prevalence [1]. Finally, elevated T2DM rates affecting Indigenous peoples [1, 7] also highlight the importance of social determinants of health [8, 9] and, in some groups, sex differences [7] in the genesis of this disorder.

Chronic kidney disease (CKD) is a serious complication of T2DM and can lead to end stage renal disease (ESRD) [10]. While ESRD affects <1% of prevalent diabetic non-Aboriginal Canadians [11], diabetes-related ESRD (DM-ESRD) – ESRD among people with diabetes caused by diabetic and non-diabetic factors – is the leading cause of ESRD in Canada, accounting for more than 35% of incident ESRD cases [12]. Importantly, diabetic Aboriginal people in Canada are at higher risk for developing DM-ESRD than their non-Aboriginal peers [11, 13, 14]. In addition, because Aboriginal people in Canada typically develop diabetes at a younger age in part because of the inter-generational impact of diabetic pregnancies experienced more frequently by Aboriginal women [5, 6], they are more likely to live long enough to transition through earlier stages of CKD and to develop DM-ESRD [15, 16].

DM-ESRD is a devastating disorder for affected people and their families, and renal replacement therapy (RRT) with peritoneal dialysis (PD), hemodialysis (HD) and renal transplantation consumes a disproportionate share of health care resources [17]. Furthermore, while the incidence of DM-ESRD in Canada has stabilized somewhat since the early 1990s, the prevalence of both T2DM and DM-ESRD continues to rise [7, 13]. Forecasting DM-ESRD numbers, while taking into account an evolving T2DM epidemic and population demographics, would allow prediction of financial, human resource and facility requirements. Moreover, simulating clinical scenarios could provide insight into how individuals and groups progress through CKD stages and health care processes, and how this influences both the health and cost burden of DM-ESRD. Accordingly, we sought to examine the potential of dynamic computer modeling [18] in better understanding the epidemiology of DM-ESRD. Our specific objectives were: 1) to develop an agent-based model that can project case numbers and treatment costs of First Nations and non-First Nations people with DM-ESRD in Saskatchewan from 1980 to 2025; and 2) to investigate the potential long term impact of simulated clinical interventions on the DM-ESRD epidemic.

## Methods

### The choice of a dynamic modeling approach

The last decade has witnessed a rapid rise in the application of dynamic models in health [18, 19]. By graphically illustrating the processes, mechanisms and clinical advances thought to be underlying changes in health outcomes and health care over time, such models support simulating scenarios to understand their effects over time on a wide variety of outcomes. Among many other uses, such models can be used to explain existing trends, project forward the status quo, investigate effects of counter-factual situations – including novel portfolios of interventions – and, via tools such as sensitivity analysis, investigate the sensitivity of projected outcomes or policy trade-offs to particular assumptions or parameter estimates. Dynamic models come in many flavours, each associated with areas of trade-off and particular contribution. For this work, we chose an agent based model (ABM) which considers the unique characteristics and evolution of individuals within study populations [20]. By preserving the heterogeneity of individuals, ABM can record and consider personal attributes (e.g. age, sex, ethnicity) and other individual characteristics (e.g. obesity) separately or in aggregate, while evaluating an individual’s interactions with other people and their geographic context. It can do so while also capturing the impact of the longitudinal progression of an individual through varying states (e.g. diabetes) throughout the life course. Accordingly, our original model was built with continuous model of time in AnyLogic 6.8.1, and was adapted to AnyLogic 7 [21] in the final phase.

### Background to study and sources of empirical data

This paper is based on a project carried out by the lead author, AG, to successfully fulfill her requirements for an MSc in Computer Science at the University of Saskatchewan (August 2013) [22]. A methodological paper was subsequently presented at the Winter Simulation Conference and published in its Proceedings [23]. In addition to published material, data for model parameters was obtained from the Canadian Organ Replacement Registry annual reports [24], Canadian Institute for Health Information special data requests [25], Saskatchewan Ministry of Health administrative data [26], as well as Saskatchewan Renal Program reports and from experts familiar with the system. Details can be found in AG’s thesis [22] and in this paper’s Additional file 1.

### Model population

The model population on which this study is based included all adults aged 20 years and older in Saskatchewan from 1980 to 2025. Saskatchewan is a mid-western Canadian province with a population that has remained at approximately one million people for several decades. First Nations people are one of three constitutionally recognized groups of Aboriginal people in Canada and currently represent about 15% of the provincial population. Within this total population, the model distinguished between four major groups in order to more accurately characterize evolution of the model population: 1) Prevalent cases with both diabetes and ESRD in 1980, 2) Prevalent cases of diabetes without ESRD in 1980, 3) Incident cases of diabetes reported between 1980 and 2005 (8275 First nations and 82,306 non-First Nations), and 4) Projected incident cases of diabetes reported between 2006 and 2025. People in Groups 1–3 were identified using administrative data [26], which also provided year of birth, ethnicity (First Nations and non-First Nations people), sex, year of diabetes diagnosis, year of ESRD diagnosis (if it occurred – 320 First Nations people and 906 non-First Nations people with diabetes developed ESRD between 1980 and 2005), and year and reason for study exit (death, end of coverage, end of study). Incident diabetes cases between 1980 and 2005 entered the model according to historic ethnicity, sex- and age-category specific yearly case counts, with the dates of diabetes diagnosis distributed uniformly throughout the incident year. Finally, incident diabetes cases in Saskatchewan from 2006 to 2025, were forecast from the previously published Saskatoon Diabetes Model [27], a System Dynamics model which can project age, ethnicity and gender specific diabetes incidence. To apply these projections to the total province, we used a scaling ratio to take into account the difference in populations between the Saskatoon Health Region and the province of Saskatchewan.

### Model structure

Four interlinked processes required representation within the model (see Additional file 1: Methods Figs. A and B for Statecharts of a person’s journey through the Saskatchewan Diabetic ESRD Model and for a person undergoing renal transplant assessment):

#### Progression from diabetes diagnosis to ESRD

Those with ESRD at the end of 1979 were placed within RRT modalities reflecting distributions drawn from CORR data from 1985 to 1989 [23]. Incident cases of diabetes occurring from January 1, 1980 onwards entered the model on the date that they received a diabetes diagnosis. Subsequently, a small proportion developed ESRD, but most lived without ESRD until death (about 55% of FN and 70% of non-FN) or study end. Since the risk of developing ESRD or dying without ESRD varies depending on time since diabetes diagnosis, CKD stage and other individual characteristics, we employed previously published parameters [15] estimated from an age, sex and ethnicity adjusted competing risks model to quantify the person-specific risk of developing ESRD or dying without ESRD at 3-year stages from diabetes diagnosis, up to a maximum category of ≥24 years.

#### ESRD treatment options and death

In the model, people with diabetes who developed ESRD were moved from their respective diabetes stages into an ESRD state, where RRT was initiated. The selection of treatments was simplified as a draw from a Bernoulli distribution which was based on the year-specific probability of receiving PD or HD as an initial treatment for people with DM-ESRD in Saskatchewan from 1981 to 2011. Bidirectional RRT transitions were incorporated between HD and PD to capture the fact that people sometimes switch between modalities. The hazard rates associated with such transitions were based on Saskatchewan data for DM-ESRD patients from 2006 to 2010. Since a newly diagnosed ESRD case occasionally receives a pre-emptive kidney transplant, the likelihood of this happening in the model was based on historic probabilities of living donor and deceased donor pre-emptive transplants.

While on dialysis, people with ESRD may be assessed for and undergo kidney transplantation (covered below). Upon receiving a transplant in the model, a person was moved from their dialysis state to a transplant state via a transition state. To capture differential graft failure, mortality and costs [28], the model recognized three post-transplant stages (≤ 90 days, 90 days-1 year, >1 year). Graft failure required a transition from the transplant state back to the dialysis state, with a hazard rate based on graft failure rates [24] stratified by transplant donor type (living or deceased) and four age groups (ages 18–44, 45–54, 55–64 and 65+). For people returning to dialysis, the dialysis modality was drawn from the same probability distribution used for people undergoing dialysis for the first time since we did not have data on dialysis modality after graft failure. The model did not consider re-transplantation without returning to dialysis.

While receiving RRT, a person’s mortality risk was calculated using a hazard function based on gender, ethnicity, age when starting treatment, type of treatment, and the length of time on the treatment. These hazard functions were derived from a risk adjusted survival analysis conducted by CIHI upon our request [24]. Because mortality hazards change with increasing age (and over time), they varied on a daily basis in the model.

#### Transplant assessment

In the model, the transplant assessment process consisted of three processes: 1) decision as to whether a transplant assessment would be carried out, 2) determination of living versus deceased kidney donor, and 3) assessment of the patient’s eligibility for transplant.

While receiving dialysis (and sometimes before dialysis is initiated particularly for those with a potential living donor), most people are sent for a transplant assessment unless older age and/or medical conditions preclude transplantation. In the model, we implemented a decision rule in which those over age 75 were not deemed suitable for a transplant, people 66 to 75 years would have a 25% chance of being assessed, and those 65 years or younger would always be assessed. Understanding that there are exceptions to these rules in reality, this decision rule was based on expert opinion from nephrologists familiar with the Saskatchewan Transplant Program.

The type of kidney transplant was important in the model because candidates for living donor and deceased donor transplantation may be on separate waiting lists, and living donors require extra evaluation, which increases costs. Type of transplant was decided early on during transplant assessment and was based on the year-specific historic proportion of living and deceased donor transplants that took place in Saskatchewan from 1981 to 1999 [24].

Determination of transplant eligibility is based on several factors, including a person’s wishes, and assessment of their physical and mental health. In the model, we considered the length of time that the assessment took, and whether the person was deemed eligible for a transplant [22]. Reflecting inter-patient variability in assessment times, an Erlang distribution function estimated the duration of the assessment by considering the number of appointments and examinations required for the individual to complete the assessment, and the average time to complete a test. We calibrated those values so that the time spent on assessment plus the time spent on the waiting list would match historical data. For the special case of patients who had received a transplant in the past year and then experienced graft failure, we assumed re-assessment within 180 days, with a mode of 90 days.

By the time that people with ESRD complete their assessment, their eligibility for transplantation is determined. In the model, we used a uniformly distributed and calibrated parameter that we called a “health coefficient” to represent a person’s overall health level, and used a calibrated cut off value of the health coefficient to determine a patient’s eligibility for a kidney transplant [22]. Those deemed suitable for a kidney transplant were then put on the transplant waiting list; others remained permanently on dialysis.

#### Transplant waiting list and kidney transplantation

People placed on a transplant waiting list can leave the list by receiving a transplant, withdrawing from the list, or dying. Priority on the waiting list is based on a number of factors, including time spent waiting, compatibility with the donor and health status. In the model, priority was randomly generated and assigned to patients when they were added to the waiting list [22]. Since earlier timing of living donor transplants (when the donor and recipient are ready for surgery) primarily depends on operating room availability, and deceased donor transplants depend on the availability of an organ, two different rates representing the frequency of living versus deceased donor transplants were used in the model.

### Model parameters and data sources

#### Mortality risks for dialysis and transplant patients

At our request, mortality risks for individuals receiving RRT were estimated by CORR using a Cox Proportional Hazards survival model conducted on ESRD patients receiving RRT in Canada from 1999 to 2008 [24]. Treatment modality, diabetes status, ethnicity, gender and age groups (every 5 years from ages 20–74 and 75 and above) were covariates. Dialysis mortality rates were further stratified by 5-year historic intervals because of improvements in survival that have occurred over the period of simulation.

#### Selection between peritoneal dialysis and hemodialysis

As noted above, the initial selection of PD or HD for incident cases of DM-ESRD depended on their historical probability. Thus, CIHI provided the count of DM-ESRD patients who received HD or PD as initial treatment in Saskatchewan from 1981 to 2011 [25]. For 1980 and the years after 2011, we used the same probabilities for PD or HD as those for the closest year for which data was available.

#### Hazard of switching between or leaving dialysis treatments

The hazard rate for switching from one type of dialysis to another was estimated using information regarding dialysis treatments for DM-ESRD patients in Saskatchewan between January 1, 2006 and December 31, 2010.

#### Graft failure rate

The estimated daily graft failure rates (hazards) used in the model were based on the graft survival curves published in 2001–2012 CORR annual reports [24]. These included the unadjusted three month, one year, three year, and five year graft survival rates for adult kidney transplant recipients stratified by age group, sex and donor type.

#### Rates of living and deceased donor transplant operations for people with DM-ESRD

These rates were estimated based on total numbers of diabetic persons receiving kidney transplants in Saskatchewan between 1983 and 2009.

#### Cost of treatment

Although a number of studies have examined the costs of ESRD treatments in Canada [17, 28–32], we used data from the Southern Alberta Transplant Program [28] that reported the costs in 2008 dollars from a payer perspective. All transplant related costs (including hospitalization costs) were taken directly from study results, while HD and PD costs were those used for comparisons (Additional file 1) in that contribution. Both discounted and undiscounted costs were projected by the model. In accordance with widespread cost effectiveness practice [33], we applied a 3% discount rate.

#### Risks of ESRD or death prior to developing ESRD

The hazards for diabetic persons developing ESRD or dying without ESRD were taken from a previously published province-wide Saskatchewan competing risks analysis by the authors covering the period 1980–2005 [15].

#### Calibrated parameters

Calibration was applied to estimate parameters lacking a reliable historical data source. The calibration process seeks to make adjustments in designated parameters so that model outputs are as close as possible to historically observed data points. Important calibrated parameters in this model included: 1) rate of withdrawal from the transplant waiting list, 2) threshold for kidney transplant eligibility 3) duration of assessment for transplantation, 4) influence of time period on the death hazard for ESRD treatment modalities, 5) pre-emptive transplant rate and type.

### Model output

Model output included metadata required to reproduce runs (model version, scenario parameters), high level statistics collected on the model population, and detailed records of an individual’s activities in the model. Such output included detailed demographic information on the simulated population of Saskatchewan people with diabetes, ESRD treatments received by individual people, information regarding those on the transplant wait list and under assessment, as well as per-patient annualized costs for treating ESRD.

### Model validation, projections and simulated clinical scenarios

To support model calibration and validation, the model was set to initiate simulation across the historic period, and to then project forward on a scenario-by-scenario basis. To enhance confidence in the model, baseline model outputs were cross-validated against several sources of historical data not used in model construction. Data was obtained from Saskatchewan Administrative databases, the Saskatchewan Renal Program, special data requests filled by CIHI, and data tables published in the CORR Annual Reports from 1981 to 2012 (see above). In some situations, DM-ESRD counts were estimated from the count for all ESRD patients. The definition of the historical data and details regarding the processing of historical data and the sources can be found in the Additional file 1. Comparisons between baseline model output and corresponding historical data were performed by visually inspecting the alignment of the trajectories created from the output values and the historical data. We ran the model with 30 realizations, each associated with a different random number sequence. We then used the statistical package R to plot the values of a given output variable from all 30 realizations onto a 2D histogram. Model output on each graph is show by multiple and primarily red bars for each year which represent different runs. Lighter colors (yellow and orange) depict a higher degree of consistency in model output.

Finally, two simulated scenarios were also run by the model using variations in model parameters and by changing relevant assumptions in the model. The first scenario examined the system-wide implications of a hypothetical advance in diabetes prevention resulting in no new diabetes cases between January 1, 2006 and Dec 31, 2025. However, people with diabetes diagnosed prior to 2006 could still develop DM-ESRD. In the second scenario, pre-emptive renal transplants were carried out on all people with diabetes when they developed ESRD. Within this second scenario, when graft failure occurred, patients spent only 90 days on dialysis prior to receiving another renal transplant. By comparing the output of these two simulated scenarios with the baseline findings, we aimed to assess the sensitivity of the model output (e.g., prevalent case count and cost) in response to the changes, and the relative impact that specific types of interventions might have on the prevalence and associated costs of DM-ESRD.

## Results

### Model validation of case numbers and renal replacement therapies

**Fig. 1:**
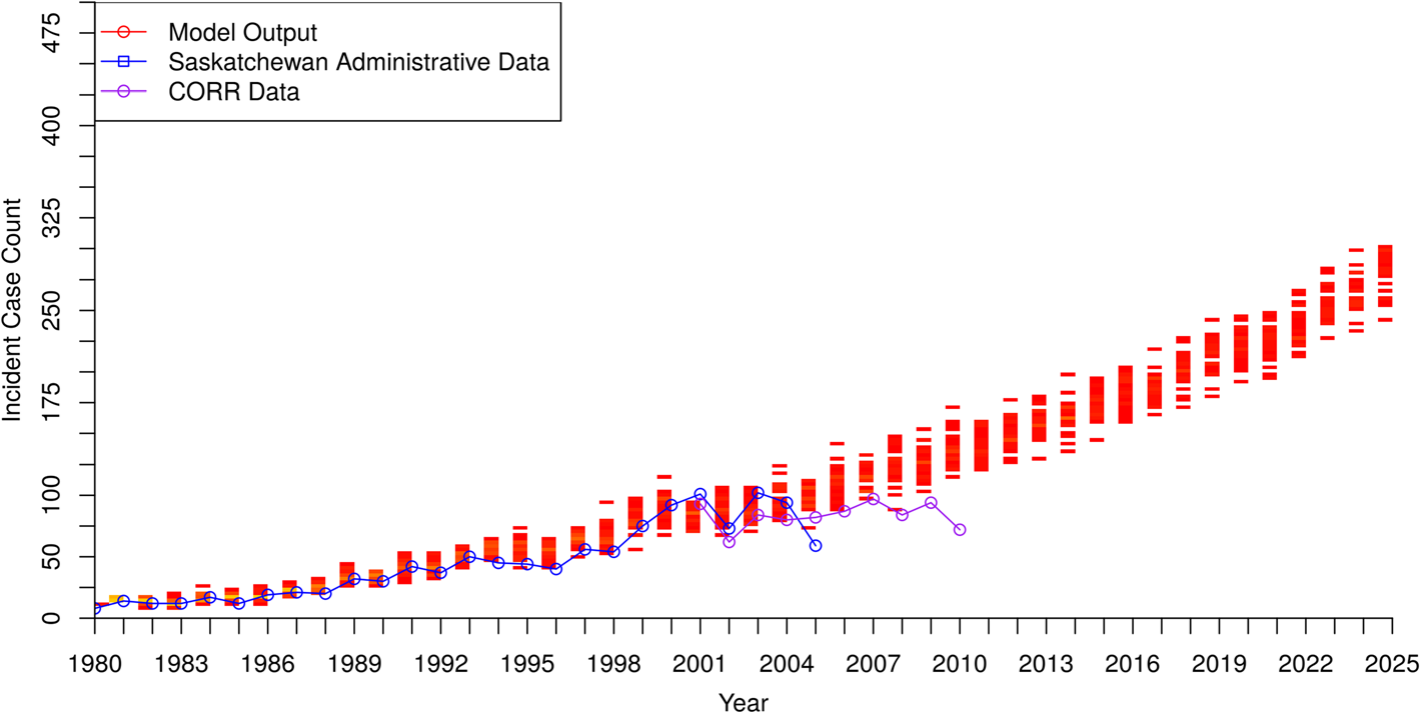
DM-ESRD Incident Case Count by Year – model output compared to historical data

**Fig. 2:**
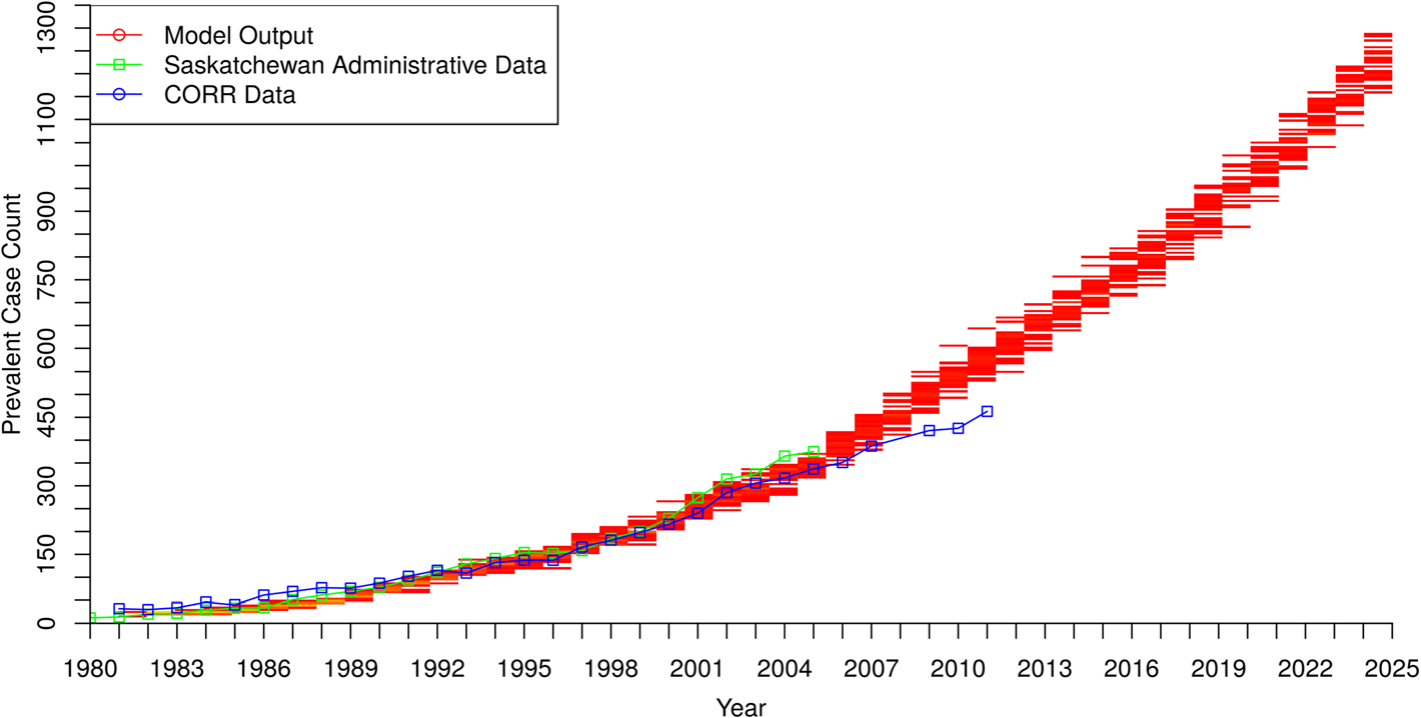
DM-ESRD Prevalent Case Count by Year – model output compared to historical data

Figures 1 and 2 show the baseline model output of incidence and prevalence of DM-ESRD in Saskatchewan beginning in 1980 and projected to 2025. For both incidence and prevalence, there was a very close match between historical data and model output until the last few years of data availability, when the curves slightly diverged. Although the model projected slightly higher case numbers in both incidence and prevalence by the early 2010’s, the overall close alignment of model output with historical data provides confidence in the model representation.

Using 2010 as the reference year, Figs**.** 1 and 2 suggest that both the incidence and prevalence of DM-ESRD will approximately double by 2025, with about 250–300 new cases per year and almost 1300 people requiring ongoing RRT by that time.

**Fig. 3:**
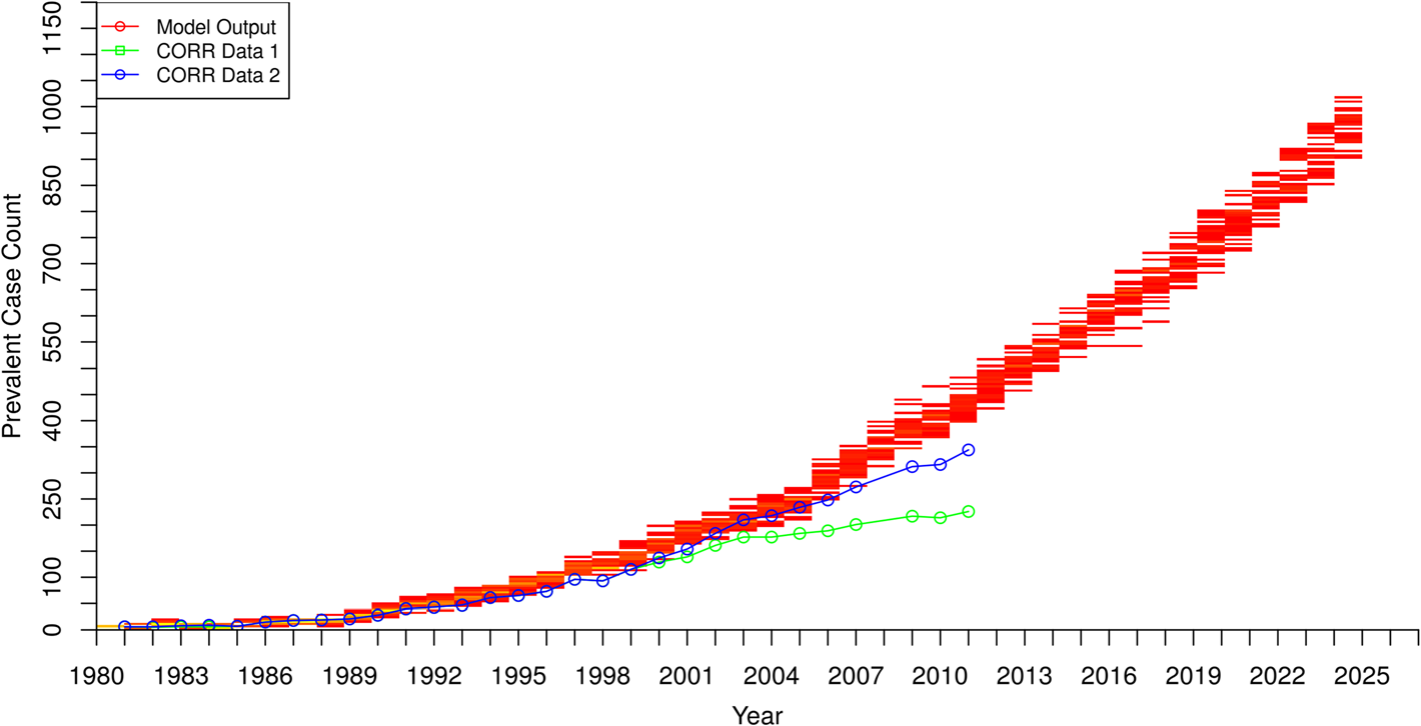
Hemodialysis Patients - Prevalent Case Count by Year – model output compared to historical data

**Fig. 4:**
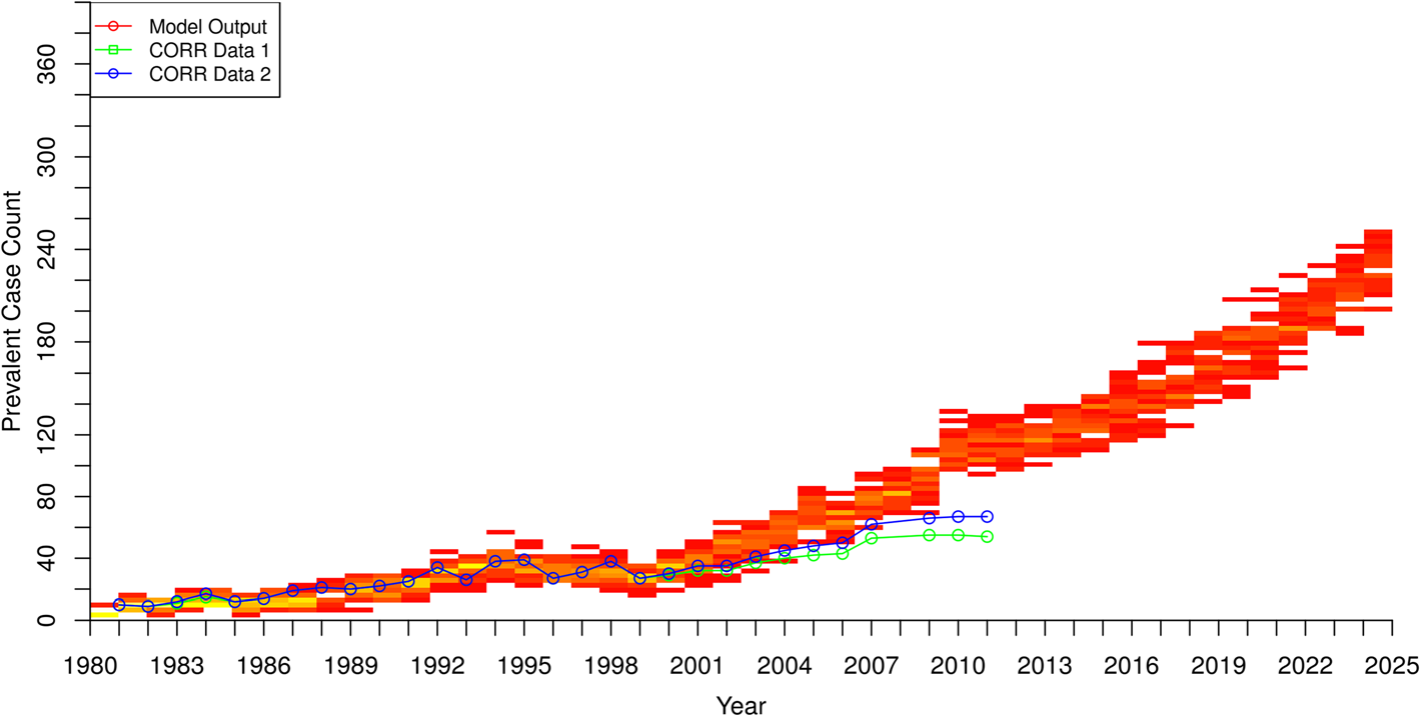
Peritoneal Dialysis Patients - Prevalent Case Count by Year – model output compared to historical data

**Fig. 5:**
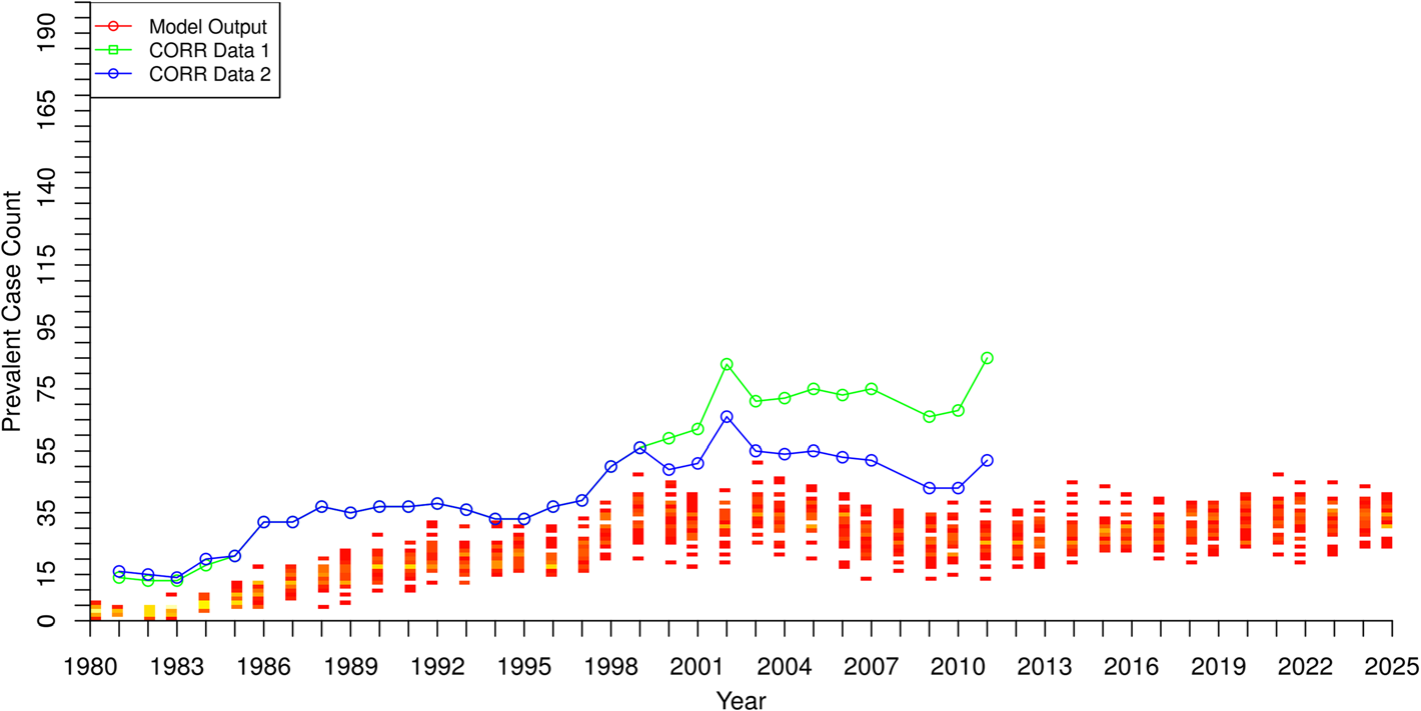
Renal Transplant Patients - Prevalent Case Count by Year – model output compared to historical data

Figures 3, 4 and 5 show the model output and historical data on prevalence of people receiving RRT with HD, PD and renal transplantation, respectively. For the first 20 years, the model output matched well with historic data for HD and PD, but after 2000 the prevalence curves generated by the model gradually diverged from historic values and overestimated the cases being treated using the two types of dialysis. In contrast, the model consistently underestimated the prevalence of renal transplant recipients by as much as 50% in the early 1980’s, with the underestimation becoming even more pronounced in the early 2000’s.

### Model projections of case numbers and costs by ethnicity

**Fig. 6:**
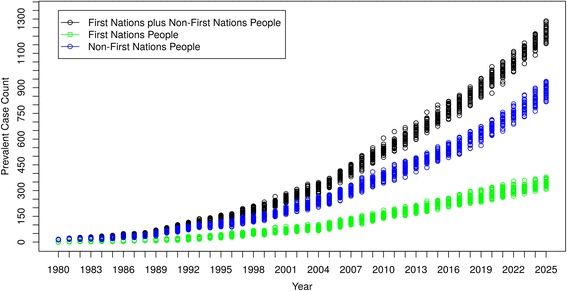
DM-ESRD Prevalent Case Count by Ethnicity

Figure 6 shows the model’s prevalent case count projections of DM-ESRD for all Saskatchewan residents and by ethnicity (First Nations people [FN], and non-First Nations people [non-FN]). The median prevalent case counts were 75 in year 1990, 231 in year 2000, 612 in year 2012 and 1229 in year 2025. The corresponding prevalent case counts for FN were 15 (1990), 62 (2000), 176 (2012) and 342 (2025). Thus, the prevalent case count for all Saskatchewan DM-ESRD patients and those in the FN subgroup more than doubled for each time interval respectively over the simulation period (with the exception of the final interval for FN, where it did not quite double). Furthermore, while FN currently constitute about 15% of the current Saskatchewan population, the prevalent case count of FN with DM-ESRD increased from 19% to 27% of all people with DM-ESRD from years 1990 to 2025.

**Fig. 7:**
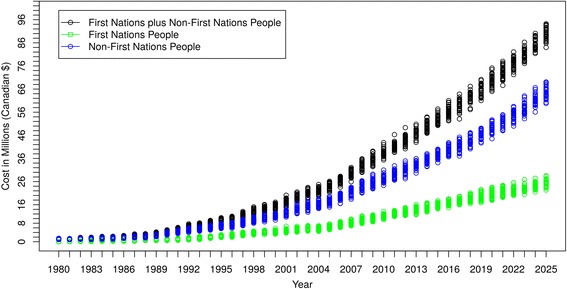
Cost/Year of Caring for DM-ESRD Patients by Ethnicity

The costs per year for treating Saskatchewan people with DM-ESRD (total and by ethnicity) are shown in Fig**.** 7. The undiscounted costs for providing services to all DM-ESRD patients in Saskatchewan were $4,311,953 in 1990, $15,613,408 in 2000, $44,576,879 in 2012, and projected to be $89,789,222 in 2025. For FN with DM-ESRD, the corresponding costs are $896,630, $4,237,449, $12,932,595 and $25,318,310, respectively. In discounted terms, the costs across both groups were $7,399,341 (1990), $19,848,532 (2000), $39,536,145 (2012), and $53,918,031 (2025). Thus, the trend in costs per year is similar to the trend found in the prevalent case count, because the cost is driven by the number of people with DM-ESRD.

### Simulated clinical scenarios

#### No new incident cases of diabetes mellitus

**Fig. 8:**
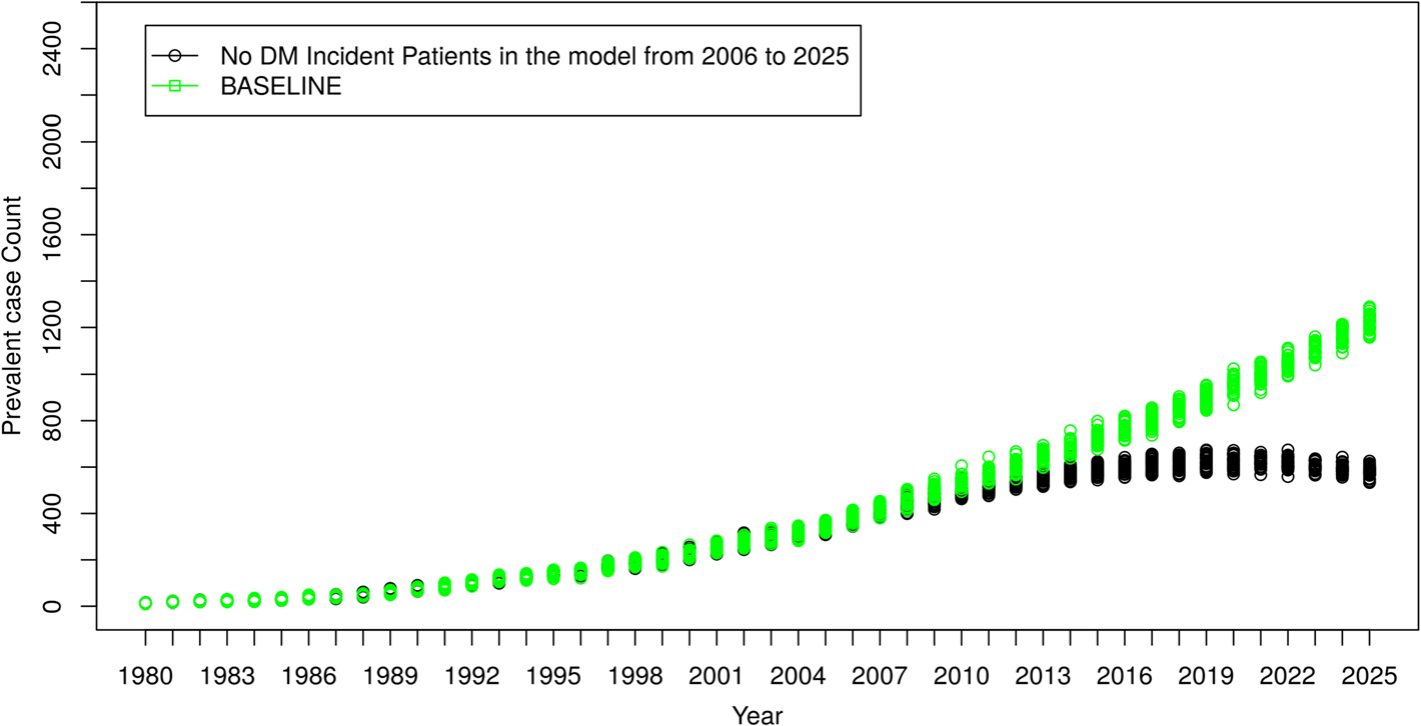
Scenario 1 – No New Incident Cases of Diabetes Mellitus after 2005

**Fig. 9:**
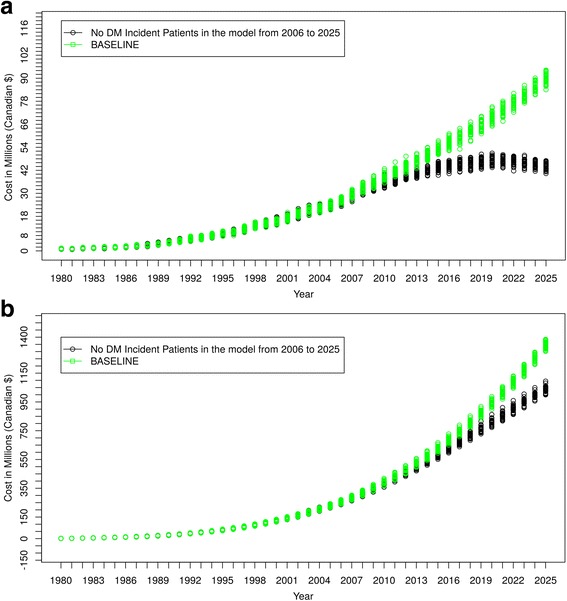
Cost Comparisons – Scenario 1 Compared to Baseline Projections. **a** Cost/Year of Caring for DM-ESRD Patients. **b** Accumulated Cost of Caring for DM-ESRD Patients

Figure 8 shows the result of the first simulated clinical scenario. Despite reducing diabetes incident cases to zero as of January 1st, 2006, the ESRD prevalent case count continued to rise for several years, and only began to decline in 2019, 13 years after new incident cases of diabetes ceased. Similar trends were also observed in the costs for caring for people with DM-ESRD (Fig**.** 9). “Baseline” curves indicate original projections of DM-ESRD and associated costs without the simulated scenario.

#### All new DM-ESRD patients received pre-Emptive renal transplant

**Fig. 10:**
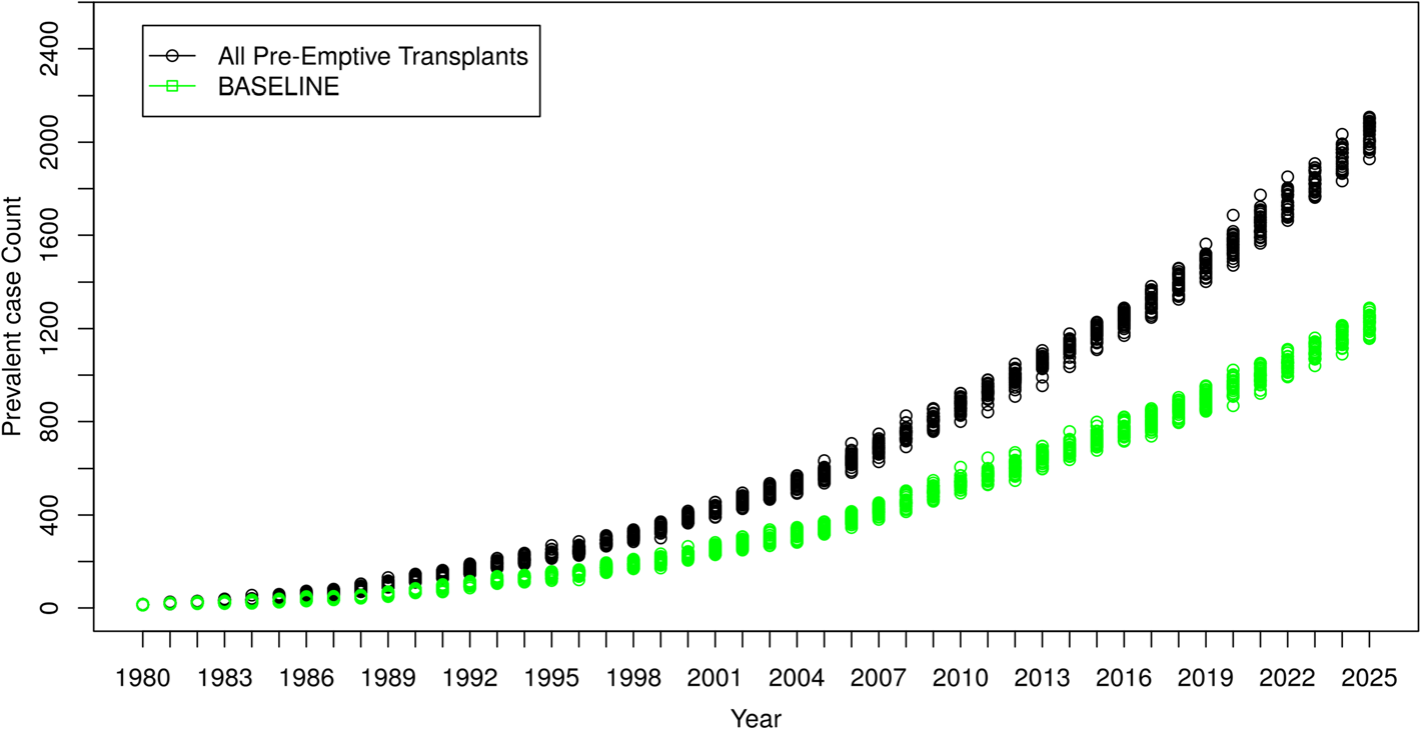
Scenario 2 - All New DM-ESRD Cases Receive Pre-emptive Renal Transplant

**Fig. 11:**
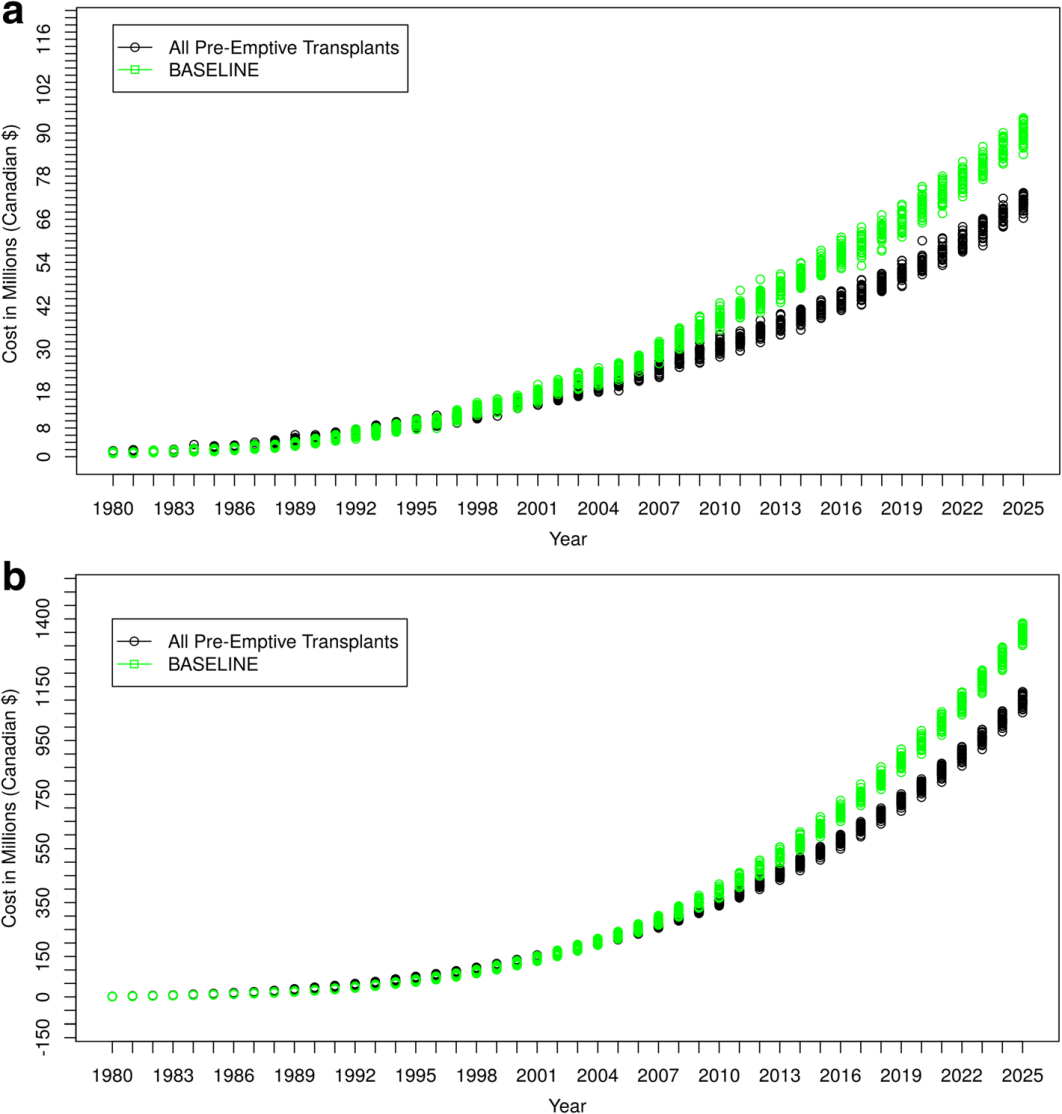
Cost Comparisons – Scenario 2 Compared to Baseline Projections. **a** Cost/Year of Caring for DM-ESRD Patients. **b** Accumulated Cost of Caring for DM-ESRD Patients

Figure 10 shows that, when all DM-ESRD incident patients received a pre-emptive renal transplant and when failed grafts were quickly replaced, there was a substantial increase in DM-ESRD prevalence compared to the model’s baseline projections, reflecting higher survival. However, despite a higher prevalence, Fig**.** 11 shows that the cost of caring for renal transplant recipients was lower than baseline projections. “Baseline” curves indicate original projections of DM-ESRD and associated costs without the simulated scenario.

## Discussion

This study describes the first agent-based model (ABM) designed to project case numbers, costs and survival of people with diabetes-related ESRD (DM-ESRD) in Canada. Using the province of Saskatchewan as its setting, this ABM closely reproduced historical trends for the incidence, prevalence and costs of DM-ESRD from 1980 to 2011, and is able to project this information into future decades. The model simulated events and activities for a population with diabetes, including year of diabetes diagnosis, progression to ESRD, type of RRT, and death. Furthermore, it considered individual patient characteristics, stages of CKD, RRT modalities, kidney transplant assessment and waiting list processes, and costs. Using this information, the model can aid in resource planning for managing the fast-growing DM-ESRD population in the province. Furthermore, the model can be used by policy makers to simulate “what if” scenarios that may provide insights into the dynamics of a diabetic person’s progression through kidney disease stages and health care processes that are otherwise not possible to achieve. Although there are two published models from Canada [34, 35] and two from the United States [36, 37] that projected various elements of case numbers, costs and complications of diabetes into the future, three of the four [35–37] used Markov models. These consider only the latest features of populations of interest and do not trace individual trajectories, or allow for calibration to or validation against longitudinal individual-level data. Importantly, none of the four models addressed ethnicity-based disparities in diabetes and its complications experienced by Indigenous peoples.

Our ABM projected a 2.5 times increase in DM-ESRD prevalence from 2012 to 2025, with First Nations people consistently accounting for approximately 30% of cases. The latter is about twice the current proportion of First Nations people within Saskatchewan’s total population. By 2025, the model projects that there will be almost 1300 prevalent cases with DM-ESRD requiring RRT in the province, with total absolute costs of almost 90 million dollars per year. Barring changes in clinical practice, close to 1000 of these individuals will be receiving hemodialysis, the most expensive form of RRT. Most of the remaining people with DM-ESRD will be on peritoneal dialysis, with fewer than 100 renal transplant recipients (see below). These projections have sobering implications not only for future RRT resource needs but also for the disproportionate demands of RRT on the provincial health care system budget [17].

While the model was particularly close to reproducing historical trends for DM-ESRD from 1980 to 2005, it displayed a modest divergence from historical data by gradually overestimating prevalent case counts from 2005 to 2011. This was primarily due to the model’s overestimation of both HD and PD patients. This overestimation of dialysis patients was partially offset by the model’s underestimation of renal transplant recipients. Thus, it appears that the model is not carrying out sufficient numbers of renal transplants among those on dialysis. We continue to examine possible factors in the model that could explain this anomaly. One possible reason is that we did not include incident diabetes cases occurring under age 20, a cohort that experiences higher transplant rates because of its young age and healthier status. This problem will be corrected in updated iterations of the model.

The ability to simulate “what if” clinical scenarios using this ABM is a rapid, powerful and economic means of projecting scenarios of interest and testing strategies to mitigate current trends. We have provided two hypothetical scenarios to illustrate the potential of this feature. While clinically implausible, these scenarios can aid in understanding the implications of more modest and plausible scenarios. In the first, we imagined that diabetes mellitus was suddenly and completely preventable. Thus, no new diabetes cases entered the model after 2005. Despite this dramatic intervention, prevalent cases of DM-ESRD (and costs) continued to rise for more than 10 years and did not begin to slowly decrease until 2019. This illustrates the tremendous inertia within the system, and the resource demands associated with caring for existing people with diabetes. It also highlights the potential value of the model in evaluating the likely impact of promising interventions.

In the second scenario, all diabetic people with newly diagnosed DM-ESRD immediately received a renal transplant, and graft losses were rapidly replaced. By 2025, this resulted in an almost 70% increase in prevalent cases of DM-ESRD but with a 30% reduction in annual costs. The increase in prevalence is due to the substantially lower mortality experienced by renal transplant recipients compared to people on dialysis (although it should be noted that an unselected group of DM-ESRD patients receiving kidney transplants would have higher mortality rates than those currently selected for transplantation and would likely also experience a decrease in graft survival). In contrast, the lower costs reflect the fact that caring for renal transplant recipients is significantly cheaper than dialysis treatment, especially following the first year of a successful transplant. Once again, while this scenario is completely unrealistic and overly simplified, it demonstrates the potential of the ABM for helping health care systems in considering different options for the allocation of health care resources.

Strengths of the model include its ability to project DM-ESRD incidence and prevalence by age, gender and ethnicity, the first model in Canada to consider all three parameters simultaneously. Second, the foundational data for the model is of high quality [24–26], and itemised cost information of RRT is taken from published research conducted in the neighboring province of Alberta [28]. Third, model output was extensively validated against historical data from multiple sources including over 25 years of longitudinal data [26]. Fourth, the current model has demonstrated its capacity for straightforward integration with other Anylogic models and an ability to incorporate a System Dynamics Model of drivers for diabetes [23]. Moreover, Anylogic software offers an animated presentation layer that allows stakeholders and other users to understand structures of the system, and provides tools to help policy makers simulate different policies and interventions in a timely manner. Finally, our Saskatchewan DM-ESRD model is equipped with a database which can record demographic information, treatment history, transplant assessment and waitlist, and cost data for every patient in the model population.

In addition to excluding people with childhood-onset diabetes, our model also has a number of limitations associated with model structures and input parameters. First, all DM incident patients in the model either developed ESRD or died before developing ESRD. However, since some patients would have instead left the province to live elsewhere, the model may have overestimated DM-ESRD incident patients. Second, the model selected transplant candidates based solely on age, whereas in reality patient selection is based on additional factors. Without considering these in the model, patients being transplanted might have died sooner or later than in reality. Third, input on cost data was taken from a Calgary study and there might be significant cost differences for managing DM-ESRD patients between Alberta and Saskatchewan. Fourth, the Cox Proportional Hazards model that was used for dialysis patients was conducted on patients from 1999 to 2008, and we adjusted the values for different time periods by adding a calendar covariate. This might have biased mortality rates for some cohorts of dialysis patients in the model. Fifth, we used a scaling factor for extrapolating the Saskatoon Diabetes Population to the Saskatchewan Diabetes Population starting in 2006. However, the proportion of First Nations people in the city of Saskatoon city might be different than the proportion of First Nations people in the province. Also, because the scaling ratio for 2006 was used for the years 2006 to 2025, it likely does not reflect population changes occurring over the later time period. Finally, many other rates used for projection are also based on years in which the values are known, and it is likely that those rates will change in the future.

## Conclusions

Over the past decade, system science methods have been increasingly applied to study problems in the public health domain to provide insights not evident using traditional approaches [18]. Dynamic models such as that presented here can capture the system wide impacts of complexity in a system that not only includes direct elements of the disease being studied but also related features such as demographics, risk factors, economic considerations, facilities and equipment, human resources, policy, budgets, and transportation. Dynamic models can be a strong learning and communication tool through visualization of diverse components in the system, and by allowing comparisons between the simulated behavior resulting from hypothesized relationships and empirical evidence. Furthermore, models can inform researchers as to which missing data could contribute the most value to decision making and understanding of system evolution. Finally, “what if” simulations using the model can help decision makers to evaluate policies and interventions that might be difficult to carry out in the real world because of ethical concerns, and time and resource constraints. Furthermore, such simulations can be used to identify the leverage points in the system and identify cost effective strategies.

In this paper, we have shown how the strengths of system science methodology can be applied to a serious public health issue in Canada by developing a dynamic agent based model of DM-ESRD. By projecting rates and costs of DM-ESRD into future decades while considering a vast array of individual characteristics, and simulating “what if” scenarios, we have shown the immense potential of this approach within a provincial health care system. While this particular project is confined to Saskatchewan, its elements and structure are adaptable and transferrable to other jurisdictions.

## Additional file

Additional file 1: Methods Fig. A: Statechart of a Person’s Journey through the Saskatchewan Diabetic ESRD Model. Methods Fig. B: Statechart of a Person Undergoing Renal Transplant Assessment. (ZIP 881 kb)

## Abbreviations

ABM: agent based model
CIHI: Canadian Institute for Health Information
CKD: chronic kidney disease
CORR: Canadian Organ Replacement Registry
DM-ESRD: diabetes-related end stage renal disease
ESRD: end stage renal disease
FN: First Nation (and non-FN)
HD: hemodialysis
PD: peritoneal dialysis
RRT: renal replacement therapy
