# Supplementary material for: Projecting prevalence, costs and evaluating simulated interventions for diabetic end stage renal disease in a Canadian population of aboriginal and non-aboriginal people: an agent based approach
Source: BMC Nephrol. 2017 Sep 4;18:283. doi: 10.1186/s12882-017-0699-y (PMC5584022; doi:10.1186/s12882-017-0699-y)
Supplement: Additional file 1: — Methods Fig. A: Statechart of a Person’s Journey through the Saskatchewan Diabetic ESRD Model. Methods Fig. B: Statechart of a Person Undergoing Renal Transplant Assessment. (ZIP 881 kb) [file 12882_2017_699_MOESM1_ESM.zip › SUPPLEMENTAL MATERIAL_ DESRD Manuscript_April 2017R1.docx]

**SUPPLEMENTAL MATERIAL**

**FIGURES**


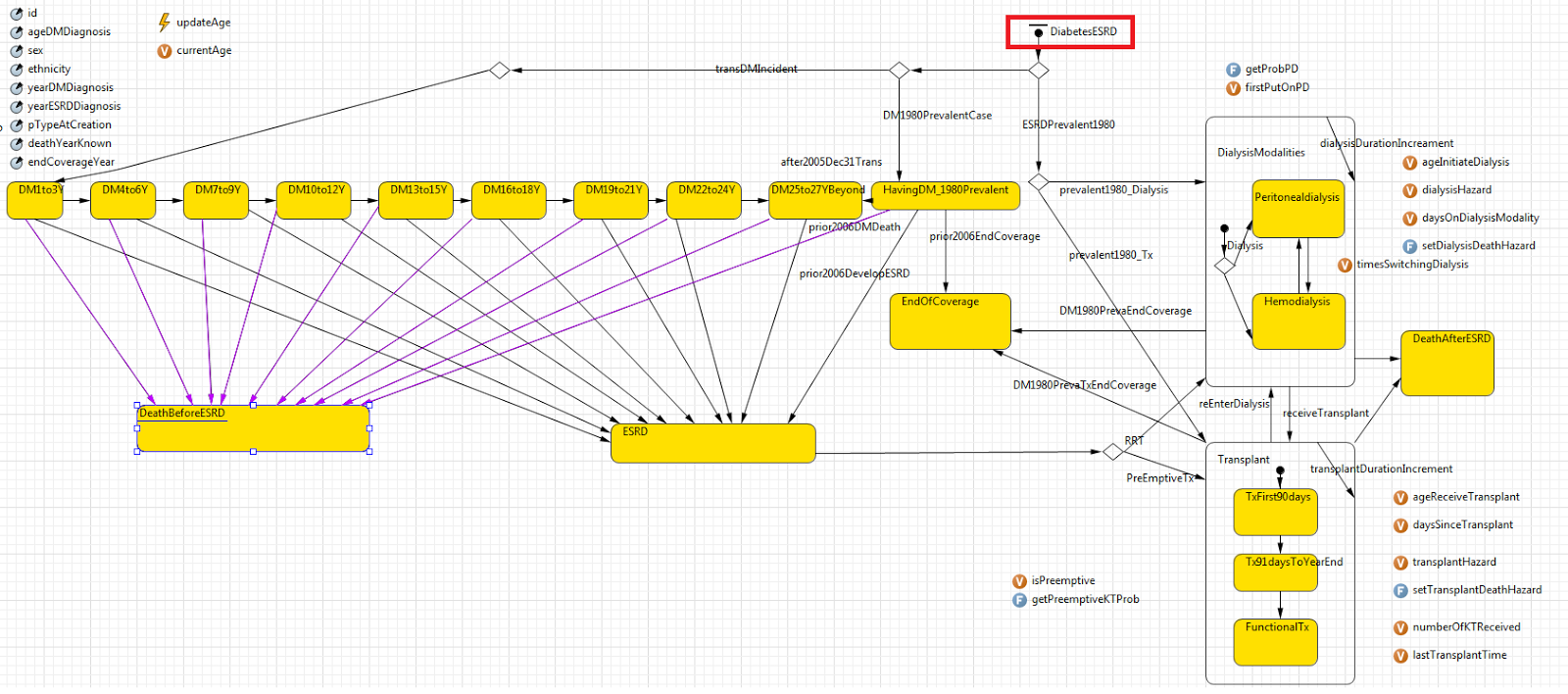


**Supplemental Figure A: Statechart of a Person’s Journey through the Saskatchewan Diabetic ESRD Model**


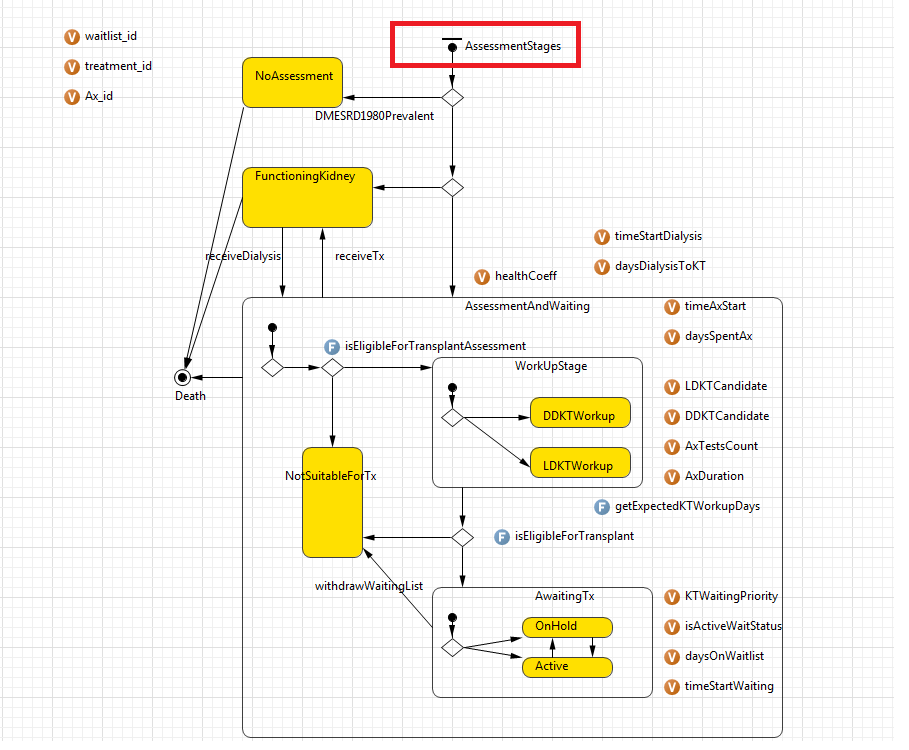


**Supplemental Figure B: Statechart of a Person Undergoing Renal Transplant Assessment**

**Appendix A – Source of Historical Data Used in Model Output Validations**

**A.1 Data from CORR, CIHI Data Requests, and Epi Study (Minimum or Zero Data Processing)**

| **Description of the historical data and file name of the historical data** | **Source** |
| --- | --- |
| The Saskatchewan DM-ESRD incident case count from 2001 to 2010.  CORR_ESRD_incident_01_10.csv | Obtained from the CORR Quick Stats – Report “Incident End-Stage Renal Disease (ESRD) Patients by Primary Diagnosis” was generated with input parameter “Primary Diagnosis=Diabetes” and “Province =Saskatchewan”. |
| The incident case count and activity case count for patients on Hemodialysis and Peritoneal Dialysis treatments between 1981 and 2011  SK_CORRRequest_PD_Activity_81_11.csv  SK_CORRRequest_PD_Incident_81_11.csv  SK_CORRRequest_HD_Activity_81_11.csv  SK_CORRRequest_HD_Incident_81_11.csv  SK_CORRRequest_TX_Incident_83_09.csv | The incident and activity case count for hemodialysis and peritoneal dialysis between 1981 and 20011 came out of the data request made to CIHI.  The data request came from the data request made to CIHI asking for the annual head counts for DM-ESRD patients who received kidney transplant between 1980 and 2011. Data from some years were removed due to confidentiality concerns. The missing data for year 2010 and 2011 were due to the suspension of kidney transplant operations in Saskatchewan. |
| The DM-ESRD prevalent case count in Saskatchewan from year 1980 to 2004  SK_ALL_Prevalent_80_05.csv | The prevalent case count for DM-ERSD patients in Saskatchewan between 1980 and 2005 was published in the DM-ESRD paper (references). The number were taken directly the “Table 4 – Prevalent ESRD” sheet in the file named “FINALtables.xls”. The original data exclude patients who die or end their coverage from the prevalent case count for a year. Also, the prevalent case count of the current year doesn’t include the incident case count of the same year. |
| The DM-ESRD prevalent case count in Saskatchewan from year 1985 to 1997 published in CORR Annual Report.  CORR_PAPER_Prevalent_TX_85_97.csv  CORR_PAPER_Prevalent_PD_85_97.csv  CORR_PAPER_Prevalent_HD_85_97.csv  CORR_PAPER_Prevalent_ALL_85_97.csv | The CORR Annual Reports from earlier years were available in paper format, in which the prevalent case count for DM-ESRD patients on different ESRD treatments from year 1985 to 1997 were given. We took the prevalent case count for HD, PD and Transplant, and add them together to obtain the total prevalent case count for DM-ESRD patients on all ESRD modalities from year 1985 to 1997. |
| The DM-ESRD prevalent case count in Saskatchewan from 2009 to 2011  CORR_DM_ESRD_Prevalent_09_11.csv | The DM-ESRD Prevalent Case count between 2009 and 2011 were given in the CORR Annual Report. (Table 17 in the 2012, 2011 and 2010 Annual Reports) |
| The annual count of death for DM-ESRD patients between 1986 and 1997.  CORR_PAPER_Death_ESRD_86_97.csv | The cumulative count of death for DM-ESRD patients between 1985 and 1997 were available in CORR annual reports. By subtracting the cumulative death count of the previous year from the cumulative death count of the current year, the death count of ESRD patients in current year can be calculated. |
| The yearly cost for care of ESRD patients |  |
| The percentage of patients who died among patients on transplant waiting list between year 2001 and 2010 in Canada.  CORR_DeathRatio_OnWaitList_01_10.csv | In the CORR annual report of 2012, the count of the patients on waiting list and the count of death on the waiting list between 2001 and 2010 are given in Table 22 - Kidney Transplant Waiting List and Deaths, December 31, Canada, 2001 to 2010. The percentage of patients who died on the waiting list is calculated. |
| The average of patients in Canada whose initial treatment is HD/PD in each year between year 1982 and 2011.  CORR_Canada_AvgAge_InitialHD_82_11.csv  CORR_Canada_AvgAge_InitialPD_82_11.csv | The data from year 1997 and before were taken from the paper copy of the CORR Annual Reports.  The data from year 2000 and 2009 were taken from the year 2011 CORR Annual Reports. The data from year 2002 and 2011 are taken from the year 2013 CORR Annual Report. |
| The average count of patients in Canada who have their first kidney transplant in each year between year 1995 and 2011.  CORR_Canada_AvgAge_GetLDKT_95_11.csv  CORR_Canada_AvgAge_GetDDKT_95_11.csv | The data for years between 1995 and 2002 were taken from Table 24 of the 2006 CORR Annual Report.  The data for years between 2002 and 2011 were taken from Table 27 of the 2013 CORR Annual Report. |
| The ratio of transplant patients returning to Dialysis over the point prevalent of transplant patients in Canada (not specific to Diabetic) in Canada  CORR_Canada_ReturnToDialysisOverYearEndPrevalent_02_11.csv | CORR report. Sheet named “return to dialysis after TX’ in validation file. |
| Withdraw from Waiting list vs Death on the waiting list | The relation between the two groups: the withdrawal rate is 1.5 to 2 times the rate of death on the waiting list  **e-Statistics Report on Transplant, Waiting List and Donor Statistics** |

**A.2 Data from Saskatchewan Health Administrative Databases**

| **Description of the historical data and the name of the file** | **Processing required** |
| --- | --- |
| Count of Death for Diabetes Patients without ESRD in Saskatchewan from 1980 to 2005  SK_DM_DeathB4ESRD_80_05.csv | The original data regarding diabetes patients in Saskatchewan from 1980 to 2005 came out of a special data request made to the Saskatchewan Administrative database in a file named “DiabeticOutcomes v3.xlsb”.  Four filters applied to the original data sources are listed as follows:   1. The age at diabetes diagnosis equal or older than 20 years old. 2. The years of receiving diabetes diagnosis includes all of the recorded years (i.e., from year 1970 to 2005). 3. The exit reason is 1, which means the reason for leaving the study is deceased.   The year of getting ESRD diagnosis is blank, which means the patients didn’t develop ESRD. |
| The Saskatchewan DM-ESRD incident case count from 1980 to 2005.  SK_DM_ESRDIncident_80_05.csv | The original data regarding diabetes patients in Saskatchewan from 1980 to 2005 came out of a special data request made to the Saskatchewan Administrative database in a file named “DiabeticOutcomes V3.xlsb”.  Three filters were applied to the original data sources, as follows:   1. The age at diabetes diagnosis equal or older than 20 years old. 2. The years of receiving diabetes diagnosis include all of the recorded years (i.e., from year 1970 to 2005). 3. Patients must be getting diabetes earlier or equal to the year of receiving ESRD diagnosis.   The details of calculations can be found in the “DMoutcome-Incident80-05” sheet of the file named “Validation_Historical_Main.xlsx”. |
| The prevalent case count for DM-ESRD patients in Saskatchewan from 1980 to 2005.  SKDMESRDPrevalent_80_05.csv | The count for DM-ESRD prevalent cases is obtained from data from a special request made to the Saskatchewan Administrative database regarding the patients living with diabetes between years 1980 and 2005 (The data on diabetes patients were in file “DiabeticOutcomes v3.xlsb”).  The prevalent case count for a given year is determined by adding the DM-ESRD incident case count of the current year to the prevalent case count from the previous year, and then subtracting the death case count of the current year. The prevalent case count formula is listed below:$P_{y} =P_{y_{-1}}+I_{y}-D_{y}- C_{y}$  P_y_ is prevalent case count for DM-ESRD patients in the current year.  P_y-1_ is the prevalent case count for DM-ESRD patients from the previous year.  I_y_ is the incident case count for DM-ESRD patients in the current year.  D_y_ is the death case count for DM-ESRD patients in the current year.  C_y_ is the count of patients who ended their coverage in the current year.  The approach used to obtain the death case count for DM-ESRD patients by year from the Saskatchewan Administrative Database is discussed in item #7 of this table.  The approach used to obtain the incident case count for DM-ESRD patients by year from the Saskatchewan Administrative Database is discussed in item #2 of this table.  The detailed calculation was recorded in the sheet “DMOutcome-Prevalent80-05” in the file named “Validation_Historical_Main.xlsx”. |
| Death Case Count of DM-ESRD Patients Per Year  SK_ESRD_Death_80_05_.csv | The yearly death count of DM-ESRD patients was obtained by applying the following filters to the diabetes patients data in file named “DiabeticOutcomes v3.xlsb”:   1. The age at diabetes diagnosis is equal or greater than 20 years old. 2. The reason for leaving the study must be “deceased”, which means the “EXITRESN” field must have value equal to 1. 3. The years of receiving diabetes diagnosis include all of the recorded years (i.e., from year 1970 to 2005). 4. The patient must have received ESRD diagnosis. The year of receiving ESRD diagnosis (“ESRDYR” field) cannot be blank. 5. Patients must develop diabetes earlier or equal to the year of receiving ESRD diagnosis. The “Diabetes before DESRD” field must have a value equal to 1. |

**A.3 Estimating the Count for DM-ESRD patients based on the Counts for ESRD Patients (including both DM-ESRD patients and other patients without DM as primary diagnosis)**

The count for DM-ESRD can be calculated by applying the proportion of DM-ESRD patients among ESRD patients to the counts for ESRD patients.

$$DMESRDCount_{y}= {DMP}_{y}\times ESRDCount_{y}$$

| **Description of the historical data and the name of the file** | **Source for all ESRD Counts** | **Source for % of DM-ESRD among all ESRD** |
| --- | --- | --- |
| The transplant incident case count for DM_ESRD in Saskatchewan between year 2001 and 2008.  ( **CORR_TX_Incident_01-08.csv** ) | The transplant incident case count for all ESRD patients in Saskatchewan between year 2001 and 2010 were obtained from the CORR Quick Stats Report “Saskatchewan Transplant by Organ Type Incidence”. | Table 27: Adult Kidney Transplant Recipients, Selected Characteristics, First Graft, Canada, 2001 to 2010 (Number, Percentage) in 2012 CORR Annual Report |
| The transplant incident case count for DM_ESRD in Saskatchewan between year 1992 and 2007.  **( SK_RenalPeport_Incident_TX_92-07.csv )** | The transplant incident case for all ESRD patients (whether DM as primary diagnosis or not) from year 1992 and 2007 in Saskatchewan are given in “the 2007 – 2008 Highlights” published by Saskatchewan Renal Program. | For year between 2001 and 2010, the proportion of patients with DM diagnosis among all ESRD patients receiving a living donor transplant and deceased donor transplant are given in the year 2012 annual report by CORR (Table 27: Adult Kidney Transplant Recipients, Selected Characteristics, First Graft, Canada, 2001 to 2010 (Number, Percentage).  For the years between 1992 and 2000, the proportions of DM-ESRD patients receiving different types of transplants were not directly given in the CORR report, but can be estimated by the forecast function in EXCEL basing the given proportions from year 2001 to 2010. |
| The incident case count for DM-ESRD starting Peritoneal Dialysis as initial treatment.  ( **CORR_PD_Incident_81_10.csv** )  **( CORR_HD_Incident_81_10.csv )** | CORR Reports from various years in both paper and online format. | Table 9 in 2012 CORR Reports  Estimation needs to be written |
| The prevalent case count for DM-ESRD patients on each treatment ESRD modality between year 2001 and 2011 in Saskatchewan.  **( SK_RenalReport_Prevalent_TX_01_11.csv )**  **( SK_RenalReport_Prevalent_PD_03_11.csv )**  **( SK_RenalReport_Prevalent_HD_03_11.csv )**  **(SK_RenalReport_Prevalent_ALL_03_11.csv)** | The prevalent case count for ESRD patients on ESRD treatments were retrieved from the Saskatchewan Renal Program reports released in year 2008 and 2012. | Between year 2008 and 2011, the proportion of DM-ESRD patients among ESRD patients living with functional transplant in the year were given in the Saskatoon Renal Program reports.  For the year between 2001 and 2007, the proportion of DM-ESRD patients among all ESRD patients living with a functional transplant was estimated based on the proportions from year 2008 to 2011 by using FORECAST function in EXCEL. |
|  |  | Between year 2004 and 2011, the proportion of DM-ESRD patients among ESRD patients receiving PD treatment in the year were given in the Saskatoon Renal Program reports.  For year 2003, the proportion of DM-ESRD patients among all ESRD patients receiving PD treatments was estimated based on the proportions from year 2004 to 2011 by using FORECAST function in EXCEL. |
|  |  | Between year 2008 and 2011, the proportion of DM-ESRD patients among ESRD patients on HD treatments in each year were given in the Saskatoon Renal Program reports.  For the year between 2001 and 2007, the proportion of DM-ESRD patients among all ESRD receiving HD treatment was estimated based on the proportions from year 2008 to 2011 by using FORECAST function in EXCEL. |
| The prevalent case count for DM-ESRD patients on ESRD treatments in Saskatchewan at Dec 31 of each year from 2001 to 2010.  **( CORR_SK_HD_Prevalent_01_10.csv )**  **( CORR_SK_PD_Prevalent_01_10.csv )**  **( CORR_SK_TX_Prevalent_01_10.csv)**  **(CORR_SK_ALL_Prevalent_01_10.csv** | The prevalent case count for all ESRD patients on HD in each year between 2001 and 2010 were obtained from the CORR quick stats by running report “Prevalent End-Stage Renal Disease (ESRD) Patients by Location of Treatment”. | *Assumed the same proportion as in estimating results saved in SK_RenalReport_Prevalent_HD_03_11.csv.* |
|  |  | *Assumed the same proportion as in estimating results saved in SK_RenalReport_Prevalent_PD_03_11.csv.* |
|  |  | *Assumed the same proportion as in estimating results saved in SK_RenalReport_Prevalent_TX_01_11.csv.* |
| The head count on the waiting list on Dec 31 of each year between 2001 and 2010.  **CORR_Waitlist_01_10.csv** | The head count of ESRD patients on waiting list between 2001 and 2010 in Saskatchewan were given in the e-statistic Report on Transplant, Waiting Lists and Donors for year 2001 and 2010. | There is no data regarding the proportion of DM-ESRD patients among ESRD patients on the waiting list. An assumption is made the proportion of DM-ESRD patients among ESRD patients on waiting list would be the same as the proportion of them for patients getting transplanted in the same year (transplant incident case count).  The proportion of DM-ESRD patients among ESRD patients getting transplanted was calculated based on data in Table 27 of the CORR annual report of year 2012. |
| **CORR_Waitlist_85_97.csv** | The head count of ESRD patients on the waiting list between 1985 and 1997 in Saskatchewan were given in the CORR annual reports (paper copies). | The proportion of DM-ESRD among ESRD patients on the waiting list between year 1985 and 1997 were estimated. An assumption was made that the proportion of DM-ESRD patients on waiting list would be the same as those getting transplanted in the same year (the same DM-ESRD% among transplant incident cases).  The percentage of DM-ESRD patients among ESRD patients receiving kidney transplant between 1985 and 1997 was calculated based on two data sources: the number of diabetes patients getting transplanted was found in the our request made to CIHI, and the total number of ESRD patients getting transplanted was found in the CORR annual reports. |
| **CORR_Waitlist_93_97.csv** | The head count of ESRD patients on waiting list between 1985 and 1997 in Saskatchewan were given in the CORR annual reports (paper copies). | The proportion of DM-ESRD patients among ESRD patients on the waiting list between 1993 and 1997 were available in CORR annual reports. |
| The operating cost by regional health authorities in operating the programs for DM-ESRD patients from 2003 to 2007.  **SK_RenalReport_Cost_03_07.csv** |  |  |

**Section A.4 Details of data processing for historical data were captured in file “Validation_Historical_Main.xlsx”.**

| **Historical Data File Name** | **Name of the Sheet in file Validation_Historical_Main.xlsx”.** |
| --- | --- |
| SK_DM_DeathB4ESRD_80_05.csv | DMoutcomes-DeathB4ESRD |
| SK_DM_ESRDIncident_80_05.csv | DMoutcome-Incident80-05 |
| **SK_DM_DeathBeforeESRD_80_05_.csv** | DMoutcome-deathAfterESRD |
| SKDMESRDPrevalent_80_05.csv | DMOutcome-Prevalent80-05 |
| SK_RenalPeport_Incident_TX_92-07.csv | SKRenalCORR DM%Tx Incident92-07 |
| CORR_TX_Incident_01-08.csv | CORR TX DM% Incident 01-08 |
| SK_RenalReport_Prevalent_TX_01_11.csv  SK_RenalReport_Prevalent_HD_03_11.csv  SK_RenalReport_Prevalent_PD_03_11.csv  SK_RenalReport_Prevalent_ALL_03_11.csv | SK_RenalReport_Prevalent |
| CORR_SK_HD_Prevalent_01_10.csv  CORR_SK_PD_Prevalent_01_10.csv  CORR_SK_TX_Prevalent_01_10.csv  CORR_SK_ALL_Prevalent_01_10.csv | CORR_ESRD Prevalent_case |
| DEATH | DEATH SHEET |
| **CORR_PD_Incident_81_10.csv**  **CORR_HD_Incident_81_10.csv** | CORR HD PD DM% Incident81-10 |
| **CORR_Waitlist_01_10.csv** | WaitlistHeadCount CORR01-10 |
| **CORR_Waitlist_85_97.csv**  **CORR_Waitlist_93_97.csv** | WaitlistHeadCount CORR85-97 |
| **CORR_PAPER_Death_ESRD_86_97.csv** | CORRPaperDeathAfterESRD86-97 |
| CORR_PAPER_Prevalent_TX_85_97.csv  CORR_PAPER_Prevalent_PD_85_97.csv  CORR_PAPER_Prevalent_HD_85_97.csv  CORR_PAPER_Prevalent_ALL_85_97.csv | CORR Paper Prevalent 85-97 |
| CORR_DM_ESRD_Prevalent_09_11.csv | CORR Report Prevalent DM-ESRD |
| SK_RenalReport_Cost_03_07.csv | SK Renal Cost |
| CORR_DeathRatio_OnWaitList_01_10.csv | CORR Waiting List Death |
| CORR_Canada_AvgAge_InitialHD_82_11.csv  CORR_Canada_AvgAge_InitialPD_82_11.csv  CORR_Canada_AvgAge_GetLDKT_95_11.csv  CORR_Canada_AvgAge_GetDDKT_95_11.csv |  |

**Appendix B – Model Output**

**Table B.1 Output Related to Diabetic Patients**

| **Statistics and Output File Name** |
| --- |
| Per-year count of DM Patients who died before developing ESRD per year  **DeathB4ESRD.csv** |
| The person years lived for overall model population per year since model start time  **PersonYearLived_DM_Yealy.csv**  The cumulative person years lived for the entire model population collected between the model start time and the end of each year.  **PersonYearLived_DM_RunTotal.csv** |

**Table B‑2 Output related to Diabetic-ESRD Patients**

| **Statistics and Output File Name** |
| --- |
| The number of new patients diagnosed with ESRD in a year.  **ESRDIncident.csv** |
| The number of patients living with ESRD (Prevalent Cases) at each year’s end.  **DMESRDPrevalent.csv** |
| The number of DM-ESRD patients who died in a year.  **DeathDMESRDPerYear.csv** |
| The person years lived for DM-ESRD patients per year since the model start time  **PersonYearLived_DMERSD_Yealy.csv**  The accumulated person years lived for DM-ESRD patients at year end since the model start time  **PersonYearLived_DMESRD_RunTotal.csv** |
| The cost for caring for DM-ESRD patients in Saskatchewan for a year  **SKYearlyTotalCost.csv**  The accumulated cost for caring DM-ESRD patients in Saskatchewan at year’s end. **SKRunningTotalCost.csv** |

**Table B‑3 Output for Patients Receiving HD**

| **Statistics and Output File Name** |
| --- |
| Count of new HD patients in a year who have never before received any ESRD treatment.  **HD_Initial.csv** |
| Count of patients who begin HD treatment in a year. Patients starting HD treatments could be returned from failed transplant, or be switching from PD treatments.  **HD_Activity.csv** |
| Count of Patients who are receiving HD treatment at the end of each year (prevalent case count)  **HDPrevalent.csv** |
| The yearly number of patients who died while receiving HD treatments in a year.  **DeathHDPerYear.csv** |
| Average age of Patients who start dialysis HD as initial treatment in the year  **AvgAgeInitialHD.csv** |
| The median of days lived from the start of HD treatment to death among people who have died in the current year and within the last four years.  **DaysLivedToDeathHD.csv** |

**Table B‑4 Output related to Patients Receiving Peritoneal Dialysis**

| **Statistics and Output File Name** |
| --- |
| Count of new PD patients in a year who have never received any ESRD treatment before.  **PD_Initial.csv** |
| Count of patients who begin PD treatment in a year. Patients starting PD treatments could be returned from failed transplant, or switching from HD treatment.  **PD_Activity.csv** |
| Count of patients who are receiving PD treatment on Dec 31 of each year (prevalent case count)  **PDPrevalent.csv** |
| Average age of patients who start PD as initial treatment in a year.  **AvgAgeInitialPD.csv** |
| The number of patients who died while receiving PD treatments in a year.  **DeathPDPerYear.csv** |
| The median of days lived from the starting of PD treatment to death among people who have died in the current year and within the last four years.  **DaysLivedToDeathPD.csv** |

**Dialysis (HD and PD)**

| **Statistics and Output File Name** |
| --- |
| Count of deaths on Dialysis (HD or PD) in a year.  **DeathDialysisPerYear.csv** |

**Transplant**

| **Statistics and Output File Name** |
| --- |
| The number of pre-emptive kidney transplants per year  **PreEmptKT.csv** |
| The yearly number of living donor kidney transplants that have taken place (incident case count). The count only includes those living donor transplants for patients who were on dialysis prior to the transplant, and excludes pre-emptive transplant.  **LDKT.csv** |
| The yearly number of deceased donor kidney transplants that have taken place (incident case count). The count only includes those deceased donor transplants for patients who were on dialysis prior to the transplant, and excludes pre-emptive transplant.  **DDKT.csv** |
| Yearly count of transplants following dialysis (incident case count)  **KT.csv** |
| Count of people living with functional transplant (prevalent case count)  **TransplantPrevalent.csv** |
| Count of Graft Failures per year  **GraftFailure.csv** |
| The number of patients who have died while receiving HD treatments in a year.  **DeathTXPerYear.csv** |
| The average age of patients when patients receive kidney transplant (first graft)  **AvgAgeInitialTX.csv** |
| The median of days lived counted from the day of the kidney transplant operation to the day of death among patients who have died in the current year and within the last four years.  **DaysLivedToDeathTX.csv** |

**Table B‑5 Output Collected on Assessment Process**

| **Statistics and Output File Name** |
| --- |
| Count of patients who weren’t sent for kidney assessment in a year.  **NotEligibleAssessment.csv** |
| Count of patients who got sent for transplant assessment in a year.  **EligibleAssessment.csv** |
| End of year count of patients who remain in the assessment process as potential candidates for a living donor or deceased donor transplant.  **HeadCountAssessment.csv** |
| The fraction of patients who passed the assessment and are deemed eligible for transplant.  **AssessmentPassRatio.csv** |
| The number of patients who have died during the transplant assessment process in a year.  **DeathDuringAX.csv** |
| Median Days spent on assessment for those patients who received transplant in this year and within the last two years. (Either living donor or deceased donor type).  **MedianDaysDDKTAXLast3Y.csv**  **MedianDaysLDKTAXLast3Y.csv** |

**Table B‑6 Output Related to Waiting List for Kidney Transplant**

| **Statistics** |
| --- |
| End of year head count for patients on the waiting list for a living donor kidney transplant.  **HeadCountOnLDKTWaitList.csv** |
| End of year head count for patients on the waiting list for a deceased donor kidney transplant  **HeadCountOnDDKTWaitList.csv** |
| End of year head count for patients on the waiting list for either the living donor or deceased donor transplant.  **HeadCountOnWaitList.csv** |
| The yearly number of patients who died while on the waiting list.  **DeathOnWaitList.csv** |
| The yearly number of patients who withdrew from the transplant waiting list.  **WithdrewWaitList.csv** |
| Median Days spent on the transplant waiting list for those patients who received a transplant (with either living donor or deceased donor type) in this year and in the last two years.  **MedianDaysLDKTWaitListLast3Y.csv**  **MedianDaysDDKTWaitListLast3Y.csv** |

**Table B‑7 Output Related to Waiting List and Assessment Process**

| **Statistics** |
| --- |
| Median number of days spent in assessment and on the wait list prior to getting transplant for those patients who received a transplant (either living donor or deceased donor type) in this year and the last two years. **MedianDaysLDKTDialysisToTXLast3Y.csv**  **MedianDaysLDKTDialysisToTXLast3Y.csv** |
| The average days spent on assessment, waiting list and dialysis (assessment and waiting list) among all patients receiving transplant in the model (over entire simulation period).  **WaitAxAvgDays.csv** |

**Appendix C - Item, Cost, Sources, Dollar Value in Original Year, and Corresponding Model Element.**

| **Cost Item** | **Original value (Year 2008 Value)** | **Model Element** |
| --- | --- | --- |
| Per-year cost for HD Patients | $83,398 | Variable named “HDperDayCost” |
| Per-year cost for PD (CAPD or CCPD) Patients | $48,472 | Variable named  “PDperDayCost” |
| Cost for Living Donor Transplant Operation | $20,108 | Variable named “LDKTOpCost” |
| Donor related cost (living donor) | $20,988 | Variable named “LDKTDonorCost” |
| Cost for caring for patients who received a living donor transplant from day 0 to the day 90 after transplant operation | $31,618 | Variable named “LDKTFirst90PerDayCost” |
| Cost for caring patients who received a living donor transplant from day 91 to day 365 | $21,932 | Variable named  “LDKT91to365PerDayCost” |
| Per-year cost for caring patients who received a living donor transplant after the 1^st^ year | $19,974 | Variable named  “LDKTAfter1stYearPerDayCost” |
| Cost for deceased donor transplant operation | $23,818 | Variable named  “DDKTOpCost” |
| Donor related cost (deceased donor) | $37,198 | Variable named  “DDKTDonorCost” |
| Cost for caring for patients who received a deceased donor transplant from day 0 to the day 90 after transplant operation | $28,200 | Variable named  “DDKTFirst90PerDayCost” |
| Cost for caring for patients who received a deceased donor transplant from day 91 to day 365 | $25,903 | Variable named  “DDKT91to365PerDayCost” |
| Per-year cost for caring for patients who received deceased donor after the 1^st^ year | $22,233 | Variable named  “DDKTAfter1stYearPerDayCost” |
