# Supplementary figures and images for: Projecting prevalence, costs and evaluating simulated interventions for diabetic end stage renal disease in a Canadian population of aboriginal and non-aboriginal people: an agent based approach
Source: BMC Nephrol. 2017 Sep 4;18:283. doi: 10.1186/s12882-017-0699-y (PMC5584022; doi:10.1186/s12882-017-0699-y)

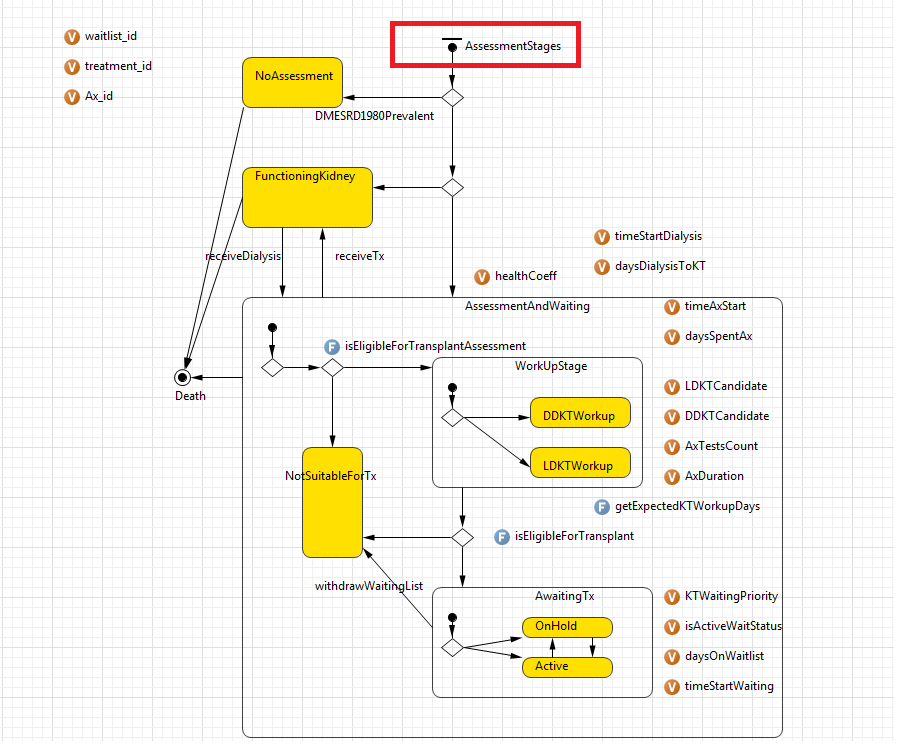

Supplement: Additional file 1: — Methods Fig. A: Statechart of a Person’s Journey through the Saskatchewan Diabetic ESRD Model. Methods Fig. B: Statechart of a Person Undergoing Renal Transplant Assessment. (ZIP 881 kb) [file 12882_2017_699_MOESM1_ESM.zip › Methods Figure BR1.png]

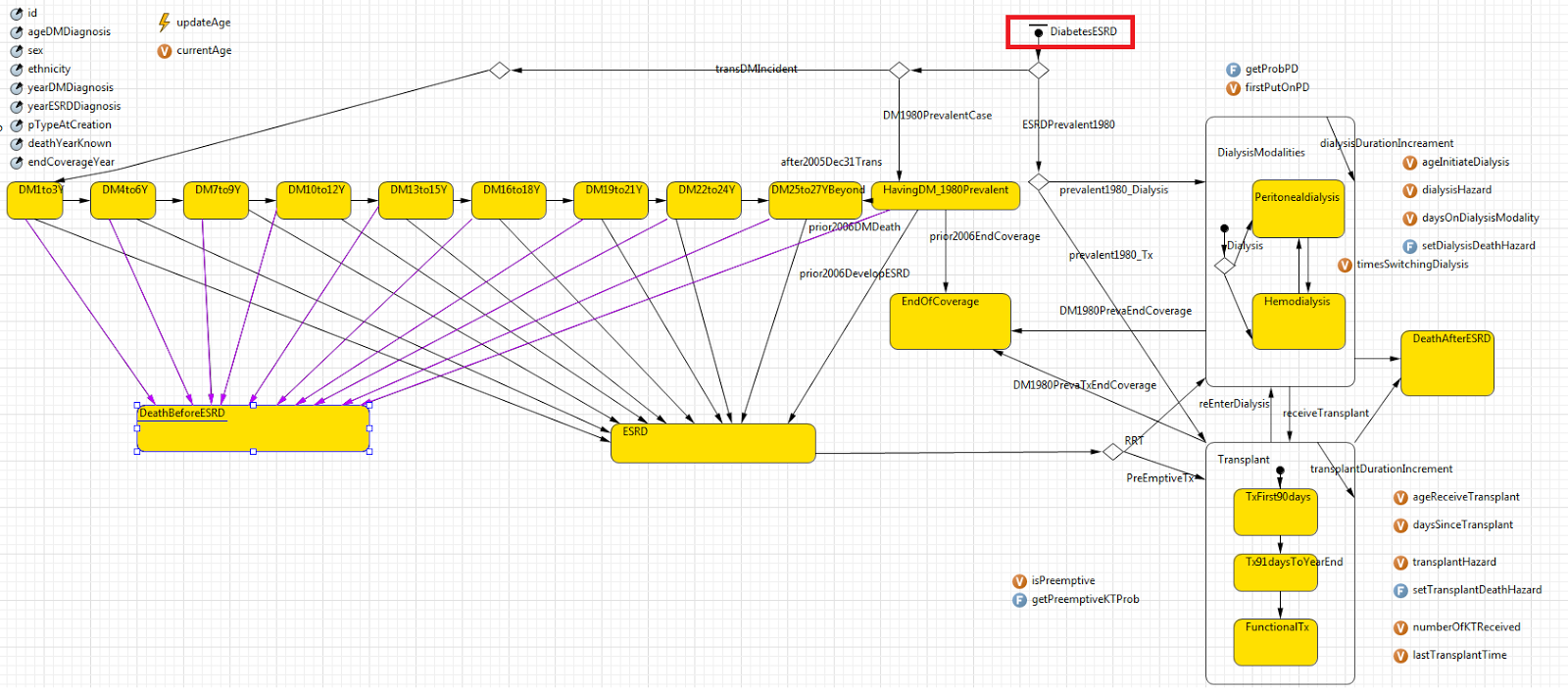

Supplement: Additional file 1: — Methods Fig. A: Statechart of a Person’s Journey through the Saskatchewan Diabetic ESRD Model. Methods Fig. B: Statechart of a Person Undergoing Renal Transplant Assessment. (ZIP 881 kb) [file 12882_2017_699_MOESM1_ESM.zip › Methods Figure AR1.png]
